# Development and characterization of microsatellite primers for *Triops granarius* (Branchiopoda: Notostraca) using MiSeq technology

**DOI:** 10.1007/s11033-022-07804-4

**Published:** 2022-09-04

**Authors:** Lina Ahmed, Yousef Al-Najjar, Emily R. A. Cramer, Gaurav Thareja, Karsten Suhre, Kuei-Chiu Chen

**Affiliations:** 1 Department of Medical Education Education City, Qatar Foundation, Weill Cornell Medicine - Qatar, 24144, Doha, Qatar; 2 Natural History Museum, University of Oslo, Sars Gate 1, 0562 Oslo, Norway; 3 Department of Physiology and Biophysics, Weill Cornell Medicine-Qatar Education City, Qatar Foundation, P.O. Box 24144, Doha, Qatar; 4 Department of Premedical Education, Weill Cornell Medicine-Qatar Education City, Qatar Foundation, P.O. Box 24144, Doha, Qatar

**Keywords:** *Triops granarius*, Microsatellite, MiSeq, Qatar, Primer development

## Abstract

**Background:**

Next-generation sequencing technology has allowed for the rapid development of microsatellites, neutral polymorphic markers that can be used for the analysis of population structure.

**Methods and Results:**

In this study, we performed whole-genome sequencing using the Illumina MiSeq system and *de novo* assembly to design microsatellite primers for *Triops granarius* populations in Qatar. The developed microsatellites are suitable for future studies of genetic structuring among geographically isolated freshwater pools. A total of 23 different primer pairs produced typical microsatellite results, with each pair successfully amplified in up to 40 individuals. Only five of the loci produced a significant departure from Hardy-Weinberg equilibrium.

**Conclusions:**

Some of the underlying mechanisms regarding the few loci that deviated from HWE may be further investigated to determine the source of deviation. As *T. granarius* is the most widely distributed species of the family, the development of these molecular markers would be useful for conducting population genetics and biogeographical studies broadly.

## Introduction

In Qatar, tadpole shrimp belonging to the genus *Triops* are recognized as the largest natural freshwater animals found in the country [1]. Specifically, *Triops granarius* has been recognized as the most widely distributed species in the regions spanning Africa to Asia [2]. Members of the species reside in temporary freshwater pools formed by seasonal rains. Since these pools are geographically isolated from one another, molecular analysis tools used to examine population structure will be of vital importance. Among several molecular tools, microsatellite markers are ideal for studying discrete populations for a variety of reasons. Unlike other highly conserved genetic markers, microsatellites are presumably neutral and demonstrate considerable polymorphism [3]. This allows for subtle differences between populations to be observed using this type of marker. Additionally, microsatellites are useful in the conservation of endangered species, thus the development of markers can support the understanding of genetic diversity in endangered species to promote better conservation practices [4, 5]. In recent years, the development of microsatellite markers has been sped up using Next-Generation Sequencing technologies [e.g., 6, 7]. In this study, microsatellite markers were developed using whole-genome sequencing and tested on samples collected from various locations in Qatar. With the development of microsatellite primers for Qatari *Triops granarius* populations, more studies can be performed in neighboring regions to investigate variation in this species.

## Methods

DNA from one individual identified as *Triops granarius* [8] collected in 2018 at the Al Khor region of Qatar (25.708694, 51.374562) was extracted using DNeasy Blood and Tissue Extraction Kit (Qiagen, #69,506). One microgram of genomic DNA was sheared to a 200–700 bp size distribution using the Covaris E220 instrument (Covaris, Inc.). The library preparation followed the manufacturer’s protocol as described in the NEBNext Ultra II DNA Library Prep kit for Illumina (Catalog No. E7645S, New England Biolabs, Inc.). Briefly, the sheared DNA was end-repaired and A-tailed to generate blunt ends, then ligated to Illumina compatible adaptors, followed by size selection using Agencourt AMPure XP Beads (Beckman Coulter, Inc.), and finally, the adaptor-ligated DNA fragments were PCR enriched. The quality of the final NGS library was assessed using the Agilent Bioanalyzer high sensitivity chip (Agilent Technologies, Inc.). The sample showed a narrow distribution with a peak size of approximately 500 bp. The sample was sequenced on the Illumina MiSeq in a paired-end 300 bp run (300 cycles). A total of ~ 49 million paired-end reads were generated for the downstream analysis.

Paired-end read data in FASTQ format was imported in CLC Genomics Workbench 12 (Qiagen, Inc.) and was quality-trimmed using CLC trimmer with read-through adapter trimming enabled. The trimmed paired-end reads were de novo assembled using De Novo assembler in the CLC Genomics Workbench (Qiagen, Inc.) with a word size of 20, bubble size of 50, minimum contig length of 100 bp, and auto-detect paired distances to perform scaffolding. The De Novo assembler algorithm works by using de Bruijn graphs. Paired reads were later remapped to contigs without updating the contigs using default parameters to calculate coverage statistics and generate mapping reports. We selected contigs with a consensus length of greater than or equal to 5,000 bp and average coverage of greater than or equal to 20X (total contigs: 5,429/164,763). Using the extract consensus sequence tool, contig sequences with coverage regions below 10X are filled with reference contig sequences. Conflicts were then resolved by voting on quality scores. The consensus contig sequences were exported in FASTA format for the identification of microsatellites. We used QDD [9] with default options of at least 5 repeats and flanking region lengths of 200 bps to identify microsatellites.

A flanking region of 100 bp was added at the start and at the end of the repeat for primer design. The flanking sequences with the repeat sequence were extracted using the bedtools v2.26.0 getfasta command [10]. The FASTA file containing the sequences was imported in CLC Genomics Workbench 12 (Qiagen, Inc.) for the primer design. In each case, the highest-scoring primer pair was reported to the lab team for validation, and for some repeats no primer pair could be successfully designed.

To efficiently test a large collection of primers in an economic manner, the forward (F) primer was concatenated with a “universal tail” (CGAGTTTTCCCAGTCACGAC, modified from [11]) within each pair of primers. A separate fluorescently tagged universal tail of the same sequence was utilized to help visualize the amplicons. These primers were applied to samples collected from locations in northern Qatar during the fall of 2017 to the fall of 2021, seven to fifteen days after rainfall. The specimens were stored in 70% ethanol in the field until dissection was conducted in the lab. A 25 mg sample was then removed from the specimen and digested at 56 degrees Celsius for 4 h to overnight followed by the DNA extraction procedure recommended by the manufacturer (Qiagen DNeasy Blood & Tissue Kit, #69,506). DNA concentration was then estimated using NanoDrop Spectrophotometer (ThermoFisher Scientific, #ND-2000) and adjusted to a standardized concentration of 15 ng/µL.

PCR was conducted on 10 µL reactions, each containing 16.5 ng of DNA, 200 nM of universal labeled F primer, 200 nM specific reverse (R) primer, 50 nM specific F primer, and 5 µL of Master Mix (*Taq* 2X Master Mix, New England Biolabs). Temperature profiles of PCR were based on [12] and consisted of denaturation at 94 °C for 5 min followed by amplification by 30 cycles at 94 °C for 30 s, continued with the annealing temperature of the specific primers (Table 1) for 30 s, then at 72 °C for 45 s. The thermal program then continued for eight cycles at 94 °C for 30 s, 53 °C for 45 s, and 72 °C for 45 s, finally ending with an elongation step at 72 °C for 10 min. Fragment analysis of amplicon was prepared by mixing 2 µL of 1:10 diluted PCR product with 0.2 µL of LIZ 500 size standards (GeneScan-500 LIZ size standard, Applied Biosystems cat#4,322,682) and 17.8 µL of Hi-Di formamide (Applied Biosystems, genetic analysis grade cat#4,311,320). Samples were analyzed using the ABI3130x Genetic Analyzer (Applied Biosystems). To validate the primers and determine the variability of the putative loci, up to 40 *T. granarius* individuals were genotyped for each primer pair.

**Table 1:** Characteristics of 23 *Triops granarius* microsatellite loci. Information for each primer pair includes the number of repeats in the representative specimen and the repeat motif, allele size range in the screened individuals (up to 40 individuals), the sample size of successfully screened individuals (N), number of alleles identified (N_A_), annealing temperature (T_M_), observed heterozygosity, (H_O_), expected heterozygosity, (H_E_), and GenBank accession number

Primer	Primer Sequence	Repeat Motif	Size (bp)	N	N_A_	T_M_ (^°^ C)	H_O_	H_E_	*P*	Accession Number
TQ1	F: 5’ - CAAAGTTTCAGCAATAGGC − 3’ R: 5’ - AAAAGGGTAGGTTGGATG − 3’	(AAT)22	195–240	32	14	54.0	0.719	0.906	0.019*	ON081520
TQ2	F: 5’ - AAATAGTCGGGCGCTGAA − 3’ R: 5’ - GGCAAACGGTCTCAAAGG − 3’	(AAT)9	153–177	26	6	57.3	0.769	0.748	0.649	ON081521
TQ3	F: 5’ - GGAACAGCCAGGAGATAG − 3’ R: 5’ - AGTGCACAGAAGAAAATCAG − 3’	(TAA)15	189–264	20	15	56.4	0.950	0.909	0.675	ON081522
TQ4	F: 5’ - CATTCATAAAACTCCCACC − 3’ R: 5’ - TGTTCCCCTCCCTTTTTC − 3’	(CAC)8	168–177	37	4	54.1	0.486	0.490	0.975	ON081523
TQ5	F: 5’ - GGTACAGGAAAGGGAGAA − 3’ R: 5’ - GAAGCAGTAAGCAAGAAGA − 3’	(TAA)15	122–158	34	12	52.7	0.824	0.872	0.630	ON081524
TQ6	F: 5’ - GCTTGAAAAAAAACGAACCC − 3’ R: 5’ - CCTGATATGTATTGCCCCT − 3’	(ATA)10	154–181	33	8	55.1	0.364	0.370	0.006**	ON081525
TQ7	F: 5’ - ATGTTTGGGTAGTGACGA − 3’ R: 5’ - GGCAAACTGGCTTTAAGA − 3’	(AAT)19	263–293	34	11	54.5	0.794	0.891	0.512	ON081526
TQ8	F: 5’ - AGATTAGTAAATTCGCGGG − 3’ R: 5’ - GGTTTTATATCGGAATGCGT − 3’	(TAA)18	267–351	39	25	55.4	0.821	0.940	0.511	ON081527
TQ9	F: 5’ -GCTGTTGTAGCATGTAGT − 3’ R: 5’ - GGAACGTTGAGGAGAAAA − 3’	(AAT)16	347–374	37	7	54.9	0.703	0.747	0.828	ON081528
TQ10	F: 5’ -CAAAGGCTACGAGAACAAA − 3’ R: 5’ - CGCCAAAAATCCCCAAAT − 3’	(CTA)12	228–252	38	4	55.3	0.632	0.507	0.608	ON081529
TQ11	F:5’ - CTACCTGCCCAAACTTCT − 3’ R:5’ - TCGCAATAAACCCTCCAC − 3’	(TTA)17	384–435	26	17	55.7	0.923	0.917	0.322	ON081530
TQ12	F:5’ - TCTCTTTGGTATTTTCCCCT − 3’ R:5’ - CACTTCACCCAACAATACTC − 3’	(TAC)12	334–355	37	7	55.4	0.595	0.648	0.813	ON081531
TQ13	F:5’ - CGCCTTTTCTTCTTGTATTC − 3’ R:5’ - TCCACTCATTCGTTACTACCT − 3’	(TTA)20	238–298	34	19	55.8	0.853	0.901	0.027*	ON081532
TQ14	F:5’ - GCGTCTCGCAATTACACA − 3’ R:5’ - AGGCAAGGAAATAGGAGT − 3’	(ATT)16	222–240	40	7	55.3	0.375	0.77	0.000***	ON081533
TQ15	F:5’ - TCAGCTCCCCCTTTACAA − 3’ R:5’ - GCCGCCTATCCTTTTATTT − 3’	(AAT)18	105–171	40	17	55.6	0.85	0.782	0.096	ON081534
TQ16	F:5’ - TGCTTGTTGACCGATGTT − 3’ R:5’ - CTGGGTTTGCTGTATCTT − 3’	(ATT)16	286–322	40	13	55.6	0.8	0.864	0.064	ON081535
TQ17	F:5’ - ACTAGACAAAAGAGCACAAC − 3’ R:5’ - CAAATCAACGCATCCTCA − 3’	(TTA)17	253–295	39	13	56.1	0.667	0.846	0.178	ON081536
TQ18	F:5’ - CTTCTTCCCTTCTTGTCTTT − 3’ R:5’ - CCTTTCTTTCGCCGTCAT − 3’	(CTA)13	232–247	38	6	56.3	0.737	0.607	0.938	ON081537
TQ19	F:5’ - TAGCAGCGTGACGAAAAA − 3’ R:5’ - CGCGGTAAGTGTAAGATT − 3’	(ATT)14	344–365	36	8	55.8	0.639	0.601	0.949	ON081538
TQ20	F:5’ - AACCATCAAACTCCTAGCCA − 3’ R:5’ - TGCCGGTTAGAGAAAGTG − 3’	(TAC)13	303–327	35	7	56.1	0.829	0.745	0.644	ON081539
TQ21	F:5’ - CGTTAAATGATGGGCGTT − 3’ R:5’ - TCCTCGTTTGGTGTTACT − 3’	(TAA)13	313–334	22	7	55.2	0.636	0.790	0.735	ON081540
TQ22	F:5’ - CATGAAACCATAAGACGCAA − 3’ R:5’ - AAAGCATCCCGAAAAAGAC − 3’	(TTA)19	199–319	39	20	55.7	0.872	0.916	0.594	ON081541
TQ23	F:5’ - CGGCGCTTTTTGTAGATT − 3’ R:5’ - GAGTGCTTGGGTGATTTTTT − 3’	(TTA)16	339–363	25	8	55.8	0.4	0.59	0.000***	ON081542

Amplicons were visualized using Peak Scanner software (version 2, Applied Biosystems) and the fragment size of each amplicon was determined. Allele names specific to the fragment sizes of the markers were established in reference to the specimen used to develop the primer sequences. GeneAlEx software (v. 6.503, [13]) was used to determine the allele frequency and the level of heterozygosity compared to predictions based on Hardy-Weinberg equilibrium (HWE).

## Results

Among 123 putative primer pairs assembled from the MiSeq contigs, 107 pairs of primers were synthesized, 94 were tested and 23 pairs were validated in 40 specimens (some specimens did not produce amplicons in some markers). Representative sequences were uploaded to GenBank (see Table 1 for accession numbers). Although some individuals did not produce amplicons for some markers, most markers produced amplicons on at least 75% of the samples. The number of alleles determined by manual counting ranged from 4 to up to 25 (N_A_). The observed heterozygosity (H_O_) ranged from 0.364 to 0.923, with expected heterozygosity (H_E_) ranging from 0.370 to 0.917. Of the 23 successful pairs of primers, five displayed a significant departure from HWE (Table 1).

## Discussion

In the screening process, an initial low success rate was experienced in a batch of 54 primer pairs, in which few primer pairs produced amplicons exhibiting typical microsatellite allele profiles. Among the handful that were successful in this batch, all showed at least eight repeats in their sequences. This prompted the employment of a new search criterion to include only those with 10 or greater repeat numbers in the screening process. This practice resulted in a larger number of primers amplifying and producing prominent peaks typical of microsatellite alleles in the chromatograms.

Analysis of the relationship between sample size and quantity of alleles yielded no distinct pattern. Among others, TQ14 successfully amplified a relatively large sample size of 40 individuals; however, it produced only seven different alleles. Therefore, an increase in sample size might not yield a wider variety of alleles.

Among 23 designed microsatellite loci, five loci (TQ1, TQ6, TQ13, TQ14 and TQ23) showed significantly departure from Hardy-Weinberg equilibrium. Several factors could have contributed to the disequilibrium observed, such as the presence of rare alleles in homozygous condition. This could have drastically impacted and skewed the distribution, resulting in a significant deviation from HWE [14]. For example, the highly significant level of genotype frequencies at locus TQ23 is most likely caused by a homozygote being the only individual with that allele. When this individual was removed from the analysis, the distribution of the genotype frequencies at this locus fell within HWE.

To understand whether the observed significant difference from HWE at TQ14 is consequent to the Wahlund effect, that is, the observation of a reduction in population heterozygosity due to subpopulation structure, samples in geographical proximity were placed in tentative clusters for analysis. Interestingly, one cluster of 16 individuals demonstrated no significant difference from HWE after grouping while the level of heterozygosity of the cluster of the remaining 24 individuals still showed significant deviation from HWE. This may indicate that the pattern of heterozygosity in the 24 individuals in the second cluster could have contributed to the deviation while not excluding any other potential causes of disequilibrium.

Other possible reasons for HWE deviation may be attributed to unequal distribution of allele frequencies across all possible alleles at any given locus due to natural selection, selective sweep, sex-linkage, inbreeding, and phenotypic preferences among individuals, or through biased sampling [15]. Among these factors, inbreeding is unlikely to have played a substantial role because the population size is large and the pattern of mating behavior in *T. granarius* appears to be random (personal observation). At least some of these potential mechanisms may be further investigated with the development of the primers in this study, which appear to show reliable patterns predicted by HWE. As *T. granarius* is the most broadly distributed species among members in the family, the use of the primers developed in this study may contribute to a greater understanding of population structure across vernal pools in Qatar, neighboring regions, and beyond.

## Declarations

### Collection permits

The collections were approved by the Qatar Ministry of Municipality and Environment MME IACUC Registration No.: MoPH-MME-003, and Assurance No.: IACUC-A-004.
